# Overexpression of NSUN2 by DNA hypomethylation is associated with metastatic progression in human breast cancer

**DOI:** 10.18632/oncotarget.10612

**Published:** 2016-11-30

**Authors:** Jie Yi, Ran Gao, Yu Chen, Zhuo Yang, Pei Han, Hui Zhang, Yaling Dou, Wenjing Liu, Wengong Wang, Guanhua Du, Yingchun Xu, Jinhua Wang

**Affiliations:** ^1^ Department of Clinical Laboratory, Peking Union Medical College Hospital, Beijing, People's Republic of China; ^2^ The State Key Laboratory of Bioactive Substance and Function of Natural Medicines, Beijing Key Laboratory of Drug Target Research and Drug Screen, Institute of Materia Medica, Chinese Academy of Medical Science and Peking Union Medical College, Beijing, People's Republic of China; ^3^ Department of Biochemistry and Molecular Biology, Beijing Key Laboratory of Protein Posttranslational Modifications and Cell Function, School of Basic Medical Sciences, Peking University Health Science Center, Beijing, People's Republic of China; ^4^ Department of Pathology, Peking Union Medical College Hospital, Beijing, People's Republic of China

**Keywords:** NSUN2, methylation, progression, biomarker, breast cancer

## Abstract

NSUN2 is a RNA methyltransferase that has been shown to be implicated in development of human cancer. However, the functional role of NSUN2, mechanism of NSUN2 overexpression and its association with clinicopathologic features in breast cancer remain unclear. To investigate alterations in the expression and functional role of NSUN2 in breast cancer, NSUN2 expression was assessed in breast cancer cells and tissues obtained from cancers at different American Joint Committee on Cancer (AJCC) stages, and its functions were investigated using breast cancer cells. NSUN2 expression was shown to be significantly higher in breast cancer cells and tissues than in normal breast epithelial cells and tissues, at both mRNA and protein levels. Overexpression of NSUN2 was shown to promote cell proliferation, migration, and invasion while NSUN2 knockdown inhibited these processes *in vitro* and *in vivo*. NSUN2 expression level was associated with the methylation level of its promoter. Our results demonstrated that the overall expression of NSUN2 significantly correlated with clinical stage (*P*=0.027), tumor classification (*P*=0.012), pathological differentiation (*P*=0.023), as well as with the expression levels of estrogen receptor (*P*<0.001), progesterone receptor (*P*=0.001), and Ki-67 (*P*<0.001). Our findings provide a unique insight into the roles and effects of NSUN2 overexpression in breast cancer cells, and highlight the necessity of the investigation of novel therapeutic targets, such as NSUN2, for the improvement of breast cancer treatments.

## INTRODUCTION

Breast cancer is by far the most common malignant diseases that affect women globally, with an incidence of 1.67 million new cases per year [1]. Although the etiology of breast cancer was previously shown to include heritable factors and genetic mutations [2, 3], molecular mechanisms underlying its development remain poorly understood. Furthermore, the clinical prognosis of breast cancer is still dependent on conventional pathologic variables, such as tumor size, tumor grade, and lymph node and distal metastasis status [4, 5]. Therefore, it is of great clinical value to further understand the molecular mechanisms underlying the progression of breast cancer and identify effective early biomarkers for the diagnosis and prognosis of the disease as well as novel therapeutic targets.

Epigenetic processes, especially DNA methylation and demethylation, play an important role in the regulation of gene expression. Since proto-oncogenes and tumor suppress genes (TSGs) maintain a dynamic balance in normal cells, and tumorigenesis is initiated and promoted by molecular abnormalities, including the activation of oncogenes and TSG inactivation [6]. Both hypermethylation of TSGs [7] and global hypomethylation [8] occur during development of cancer. Aberrant promoter CpG island hypermethylation of TSGs can lead to transcriptional silencing and result in tumorigenesis [9, 10], while hypomethylation was proposed to result in the activation of proto-oncogenes, such as MYC or HRAS [11–13].

NSUN2 (NOP2/Sun domain family member 2; MYC induced SUN domain-containing protein, Misu) is a RNA methyltransferase implicated in cell proliferation [14, 15], stem cell differentiation [16], testis differentiation [17], and human cancer [14, 18]. TRNA, mRNA, and microRNA can be methylation substrates for NSUN2 [14, 19–22]. Our previous study showed that NSUN2 promotes cell growth through the methylation of CDK1 mRNA, stabilizing it and increasing the level of translation [21]. NSUN2 that methylates RNA pol III transcripts was identified as a *MYC* target gene, necessary for *MYC*-induced cell growth and proliferation [14]. This gene, similar to MYC, is overexpressed in human and animal cancers, such as squamous cell carcinoma and colorectal cancer. However, the functional role of NSUN2 in breast cancer tumorigenesis and clinical staging remains unknown.

In this study, we firstly report NSUN2 expression levels in breast cancers of different clinicopathological grades, and show that NSUN2 expression correlates with the clinical staging, tumor classification, and pathological differentiation. Additionally, we show that NSUN2 expression was associated with estrogen receptor (ER) or progesterone receptor (PR) and Ki-67 in breast cancer. Mechanistically, our results showed that DNA hypomethylation leaded to the overexpression of NSUN2. Taken together, the results presented here strongly suggest that NSUN2 plays an important role in development and progression of breast cancer, and that NSUN2 may be a novel therapeutic target.

## RESULTS

### NSUN2 mRNA is overexpressed in breast cancer tissue samples and its levels are negatively correlated with ER status

**Figure 1:**
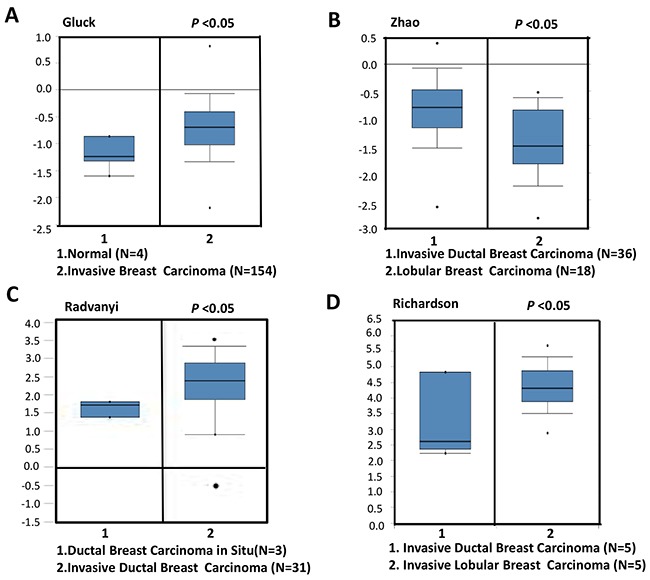
NSUN2 levels in invasive breast cancers versus normal breast tissues or breast cancers in situ, according to the results found in Oncomine database **A**. NSUN2 levels in invasive breast cancers vs normal tissue samples. **B**. NSUN2 expression in invasive ductal breast cancers vs lobular breast carcinomas. **C**. NSUN2 expression levels in invasive ductal breast cancers vs ductal breast cancers in situ. **D**. NSUN2 levels in invasive lobular breast cancers vs invasive ductal breast cancers.

**Figure 2:**
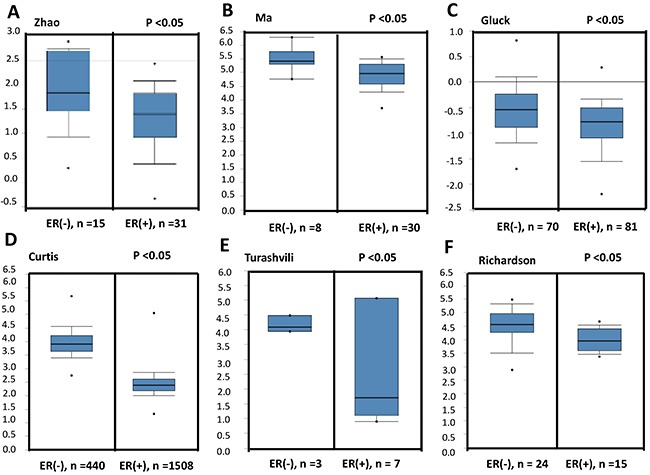
NSUN2 expression in ER-negative or positive breast cancers, according to the datasets found in Oncomine database **A-F**. NSUN2 expression in ER-negative breast cancers was shown to be higher than that in ER-positive breast cancers using datasets fromwww.oncomine.org (Zhao et al.,2004; Ma et al., 2009; Gluck et al.,2011; Curtis et al., 2012; Turashvili et al., 2007, Richardon et al., 2006).

To confirm the relationship between NSUN2 expression levels and breast cancer progression, we compared the expression levels of NSUN2 in normal tissues and breast cancer tissues using Oncomine database, which provides publicly available cancer gene expression datasets (www.oncomine.org). The results showed that NSUN2 mRNA expression levels were upregulated in the invasive breast cancer tissues, compared with the normal tissues (Figure 1A), and this is especially true for invasive lobularl breast carcinomas, compared with ductal breast carcinomas (Figure 1B and 1D), as well as invasive ductal breast carcinoma vs ductal breast carcinoma in situ (Figure 1C). Real-time PCR analysis, using 10 pairs of matched breast cancer and normal tissues, showed that NSUN2 was overexpressed (*P*<0.05) in 100% (10/10) of breast cancer patients, which is consistent with the data obtained from Oncomine database (Supplementary Figures 1 and 2A). The development of breast cancer is generally associated with hormone receptor levels, and therefore, we further examined the relationship between NSUN2 expression and ER status in breast cancer tissues. We compared NSUN2 expression levels and ER status using Oncomine datasets of gene chip profiles classified by ER positivity. As shown in Figures 2A-2F, we observed a significant negative association between NSUN2 expression levels and ER positivity (*P*<0.05). Together, all these results suggested that NSUN2 was related to progression of breast cancer.

### NSUN2 is upregulated in human breast cancer cells and tissues

**Figure 3:**
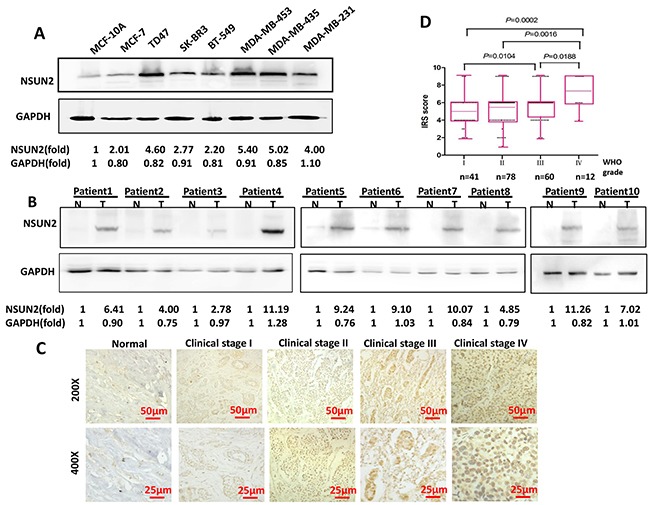
NSUN2 expression in breast cancer cells and tissues **A**. NSUN2 protein levels in normal breast epithelial cell (MCF-10A) and in seven breast cancer cell lines. GAPDH was used as a loading control. **B**. NSUN2 protein levels in 10 matched primary breast tumor tissue samples (T) and adjacent non-tumor tissues (N) obtained from the same patient. **C**. NSUN2 expression levels in normal and tumor breast tissues (n=191) at indicated stages (stage I, n=41; stage II, n= 78; stage III, n= 60; and stage IV, n= 12). Original magnification, 200× and 400×; scale bars, 50 μm and 25 μm, respectively. **D**. NSUN2 protein expression immunoreactive score (IRS) in patients in four clinical stages. IRS increased with the clinical stage (stage II, *P*=0.0016; stage III, *P*=0.0104; stage IV, *P*=0.0002). Results from Western blot are representative of three independent experiments and the signals were quantified by densitometry.

To validate the expression of NSUN2 in breast cancer cells and tissues, we examined the protein levels of NSUN2 using Western blot. It was shown in Figure 3A that expression of NSUN2 was significantly upregulated in seven breast cancer cell lines compared to MCF-10A. Similarly, Western blot and IHC both showed that NSUN2 protein was upregulated in breast cancer tissues compared to their matched adjacent non-tumor tissues from the same patient (Figure 3B and 3C). The mRNA levels of NSUN2 in six breast cancer cell lines were also higher than that in MCF-10A (*P*<0.05) (Supplementary Figure 2B). Taken together, these results indicated that there was overexpression of NSUN2 in breast cancer.

### Methylation of NSUN2 promoter CpGs in breast cancer cells

**Figure 4:**
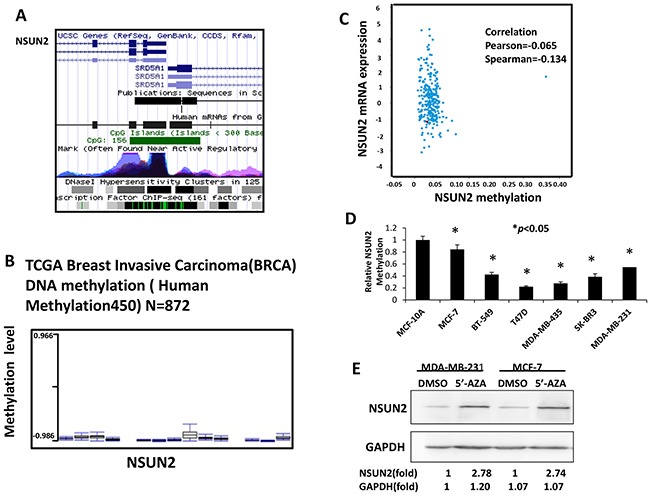
NSUN2 promoter CpG island is methylated in breast cancer cells **A**. CpG island (156 bp) was found in NSUN2 promoter region. **B**. NSUN2 methylation level in breast cancer cells. **C**. TCGA database-derived data show there was the low methylation of whole NSUN2 DNA and high NSUN2 mRNA expression in tissues in breast cancer. **D**. NSUN2 methylation level in six breast cancer cell lines, relative to normal breast epithelial cell (MCF-10A) methylation level. E. NSUN2 expression in MDA-MB-231 and MCF-7 cells treated with 5′-AZA, compared with the expression in cells treated with DMSO. Real-time qPCR data was obtained in three independent experiments, and the results are presented as mean ± standard deviation (SD) of three independent experiments; *P<0.05. Results from Western blot are representative of three independent experiments and the signals were quantified by densitometry.

More and more studies have showed that gene expression was regulated by epigenetics, especially through methylation of gene promoter regions. Silicon analysis was done using UCSC gene browser (http://genome.ucsc.edu) and TCGA database (http://www.cbioportal.org/public-portal/cross_cancer.do). As illustrated in Figure 4A, there is a 156bp CpG island in the promoter region of the NSUN2 gene. Results from TCGA data analysis showed that there was significantly low methylation of NSUN2 in breast cancer tissues (Figure 4B), and that there was the low methylation of whole NSUN2 DNA and high NSUN2 mRNA expression in tissues in breast cancer (Figure 4C). Therefore, to explore mechanism of NSUN2 overexpression in breast cancer, we assessed methylation level of NSUN2 gene promoter in six breast cancerous and one non-cancerous cell lines. Methylation levels NSUN2 promoter in six malignant cell lines (MDA-MB-231, MCF7, MDA-MB-435, BT549, SK-BR3, and T47D) were significantly lower (*P*<0.05) than that in MCF-10A cells (Figure 4D). To explore if the methylation of NSUN2 affects its expression, we treated the MDA-MB-231 and MCF-7 cells, the expression levels of NSUN2 of which were low, with 5′-AZA (5-azacytidine) and control (DMSO). The results showed that NSUN2 expression in MDA-MB-231 and MCF-7 cells treated by 5′-AZA was increased compared with control, respectively (Figure 4E). Overall, these results suggested that NSUN2 was epigenetically overexpressed due to DNA hypomethylation in breast cancer cells.

### NSUN2 promotes proliferation of breast cancer cells

**Figure 5:**
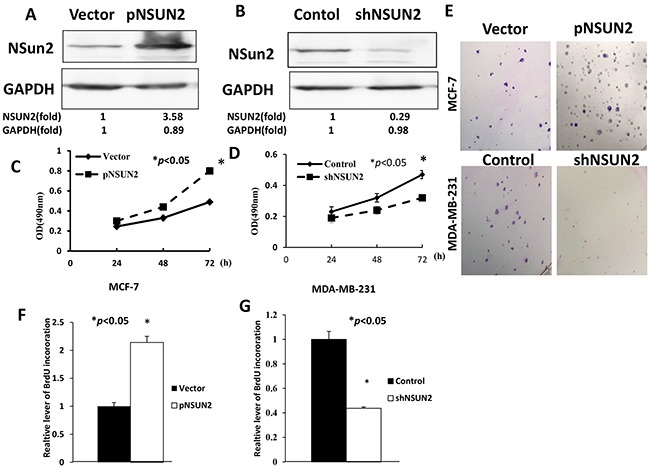
NSUN2 promotes proliferation of breast cancer cells **A**. NSUN2 expression in MCF-7 cells stably infected with lentiviral particles carrying pHBLV-NSUN2 vector (pNSUN2). **B**. NSUN2 expression in MDA-MB-231 cells stably infected with lentiviral particles carrying pHBLV-shNSUN2 vector (shNSUN2). **C** and **D**. After transfection, cell proliferation was determined by CCK-8 assay. OD, optical density. **E**. Colony formation assay results. **F** and **G**. BrdU incorporation leveldetermined by BrdU-DNA synthesis assay. The data were obtained in three independent experiments. All values are given as mean ± standard deviation (SD) of three independent experiments; *P<0.05.

To explore the functional role of NSUN2 in breast cancer, stable overexpression of NSUN2 in MCF-7 cells was established by the subcloning of full-length human NSUN2 cDNA into the pHBLV vector (pHBLV/NSUN2) (Figure 5A) and stable MDA-MB-231 cells which have low NSUN2 expression by NSUN2 shRNAs (pHBLV/shNSUN2) was obtained (Figure 5B). CCK-8 assay showed a significant increase in the number of NSUN2-overexpressing MCF-7 cells compared with the control after 72 h of culture (*P*<0.05) (Figure 5C), indicating that NSUN2 overexpression increases the proliferation of breast cancer cells. Colony formation assay showed similar results (Figure 5E, top panel). Conversely, NSUN2 knockdown led to a decrease in the growth of MDA-MB-231 cells (*P*<0.05) (Figure 5D), which was confirmed by the colony formation assay as well (Figure 5E, lower panel). BrdU cell proliferation assay was performed to assess the proliferation of MDA-MB-231 and MCF-7 cells, and the results showed that the percentage of BrdU positive cells was significantly increased (*P*<0.05) in NSUN2 overexpressing cells and significantly decreased (*P*<0.05) in NSUN2-knockdown cells (Figure 5F and 5G). All in all, these results confirmed that NSUN2 promoted proliferation of breast cancer cells.

### NSUN2 promotes migration and invasion in breast cancer cells

**Figure 6:**
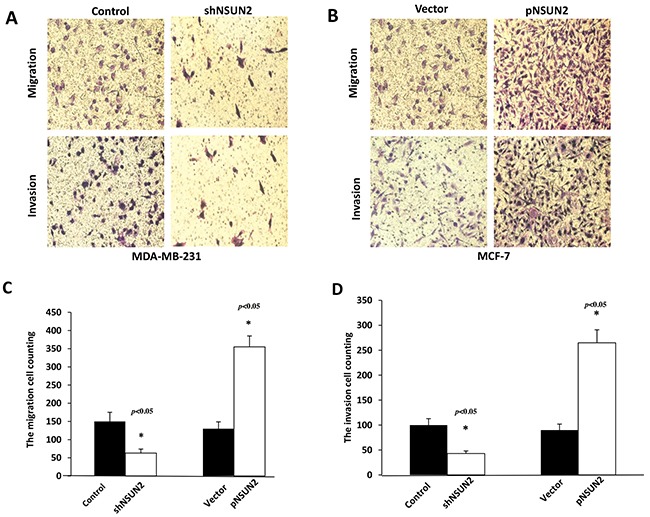
NSUN2 expression promotes migration and invasion of breast cancer cells **A** and **B**. Representative photographs of the effects of NSUN2 knockdown in MDA-MB-231 and overexpression in MCF-7 cells, respectively, obtained using cell migration and invasion assays. **C** and **D**. Giemsa-positive cells were identified using light microscope (200×). The data are represented as mean ± standard deviation (SD), obtained in three independent experiments; *P<0.05.

To investigate if NSUN2 promotes metastasis and invasion of breast cancer, we performed migration assays and invasion assays in breast cancer cells. As presented in Figure 6A and 6C, NSUN2 knockdown led to a decrease in MDA-MB-231 cell migration (mean 63 ± 11.2%; *P*<0.05), while the overexpression of NSUN2 led to an increase in MCF-7 migration (mean 355 ± 29.6%; *P*<0.05). NSUN2 knockdown led to a decrease in MDA-MB-231 cell invasion rate (mean 43 ± 5.2%; *P*<0.05), while NSUN2 overexpression led to the increase in MCF-7 cell invasion (mean 265 ±26.1%; *P*<0.05) (Figure 6B and 6D). Therefore, the results show that NSUN2 does increase cell motility.

### NSUN2 expression is associated with clinical features in breast cancer

To investigate the clinical significance of the upregulation of NSUN2 in breast cancer, we analyzed the potential associations between NSUN2 expression and the clinical characteristics of paraffin-embedded breast cancer tissue specimens obtained from 191 patients, including 41 (21.5%) stage I cases, 78 (40.8%) stage II cases, 60 (31.4%) stage III patients, and 12 (6.3%) stage IV cases. IHC analyses showed that 54 of 60 (90.0%) ER-negative paraffin-embedded breast cancer tissue samples displayed moderate or strong NSUN2 nuclear staining, whereas 56 of 131 (42.7%) ER-positive tumor demonstrated a decreased rate of NSUN2 staining (*P*<0.001). Similarly, 59 of 72 (81.9%) progesterone receptor (PR)-negative paraffin-embedded breast cancer tissues displayed moderate or strong nuclear NSUN2 staining, whereas 49 of 119 (41.2%) PR-positive tumor samples showed a decrease in NSUN2 staining (*P*=0.001). As shown in Table 1, detailed analyses demonstrated that the expression of NSUN2 is significantly associated with clinical stage (*P*=0.021), tumor (T) classification (*P*=0.012), pathological differentiation (*P*=0.023), ER status (*P*<0.001), PR status (*P*=0.001), and Ki-67 expression level (*P*=0.008). Immunoreactive score of Remmele and Stegner (IRS) statistics for NSUN2 are presented in Figure 3D. IRS was shown to increase with clinical stage (stage II, *P*=0.0016; stage III, *P*=0.0104; stage IV, *P*=0.0002). NSUN2 intensity in all groups was analyzed by Image J software, which also showed that NSUN2 protein expression was shown to be significantly increased in tumor stages I-IV (stage I, P=0.04; stage II, P=0.005; stage III, P=0.017; stage IV, P=0.005)(Supplemental Figure 3). However, it was not shown to be associated with patient age, node (N) classification, metastasis (M) classification, and HER2 expression. Spearman association coefficients between NSUN2 expression and clinical stage, Tumor classification, ER status, PR status, pathological differentiation, and Ki-67 expression were 0.217 (*P*=0.003), 0.216 (*P*=0.003), -0.325 (*P*<0.001), -0.239 (*P*=0.001), -0.158 (*P*=0.034), and 0.25 (*P*<0.007), respectively (Table 2). Taken together, these results indicated that expression of NSUN2 was associated with many key clinical features of breast cancer.

**Table 1:** Clinicopathologic characteristics of patient samples and expression of NSUN2 in breast cancer and association between NSUN2 expression and clinicopathological characteristics of breast cancer patients

Characteristics	NSUN2		*P*
	Low or no	High	
**Age(y)**			0.192
≤53	37	64	
>53	25	65	
**Clinical stage**			0.021
I	20	21	
II	26	52	
III	15	45	
IV	1	11	
**T classification**			0.012
T1	33	37	
T2	24	78	
T3	3	9	
T4	2	5	
**N classification**			0.12
N0	36	55	
N1	11	20	
N2	6	22	
N3	9	32	
**Distant metastasis**			0.065
Yes	1	11	
No	61	118	
**ER**			< 0.001
Positive	56	75	
Negative	6	54	
**PR**			0.001
Positive	49	70	
Negative	13	59	
**HER2**			0.308
Positive	52	100	
Negative	10	29	
**Pathological differentiation**			0.023
Well differentiated	5	12	
Moderately differentiated	41	63	
Poor differentiated	11	48	
**Ki-67**			< 0.001

**Table 2:** Spearman association analysis between NSUN2 and clinical pathologic factors

Variables	NSUN2 expression level	
	Spearman association	*P*-value
Clinical staging	0.217	0.003
T classification	0.216	0.003
ER	-0.325	< 0.001
PR	-0.239	0.001
Pathological differentiation	-0.158	0.034
Ki-67	0.25	< 0.001

### NSUN2 promotes the tumorigenicity of breast cancer cells *in vivo*

**Figure 7:**
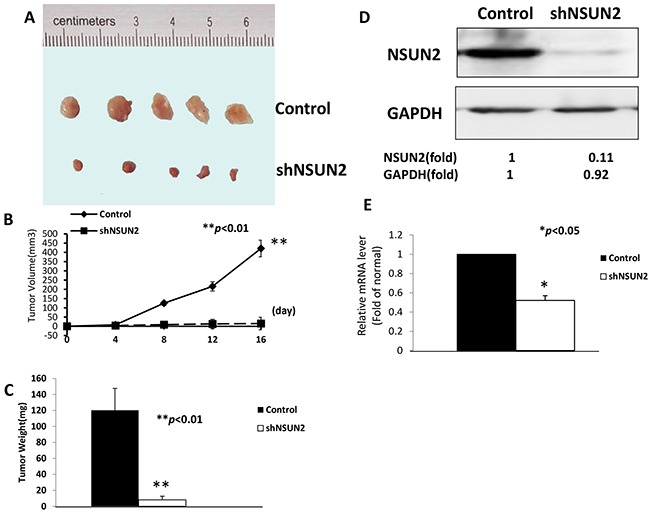
NSUN2 overexpression contributes to the progression of breast cancer *in vivo* Xenograft nude mouse model were inoculated with MDA-MB-231 shNSUN2 cells or the cells carrying control vector (n=5/group), at day 16 after inoculation. **A**. Tumors isolated from the control mice and the mice inoculated with MDA-MB-231 shNSUN2 cells. **B**. Xenograft tumor growth curves. **C**. Mean weights of xenografts tumors. **D** and **E**. Expression levels of NSUN2 protein and mRNA in xenograft tumors were shown. The data were obtained in three independent experiments. All values are given as mean ± standard deviation (SD) of three independent experiments; *P<0.05. Results from Western blot are representative of three independent experiments and the signals were quantified by densitometry. *P<0.05,* *P<0.01.

To determine whether NSUN2 expression promotes tumorigenicity in breast cancer cells *in vivo*, we established a xenograft model by inoculating nude mice (n=5/group) with MDA-MB-231/shNSUN2 or empty vector. The growth of the tumors formed by NSUN2-knockdown cells was significantly smaller (Figure 7A and 7B) (*P*<0.01), and had lower mass (Figure 7C) (*P*<0.01) than those formed by the cells transfected with the empty vector. Western blot and qRT-PCR analyses showed that NSUN2 protein and mRNA levels in tumors formed by NSUN2-knockdown cells are lower than those in the control cells (*P*<0.05) (Figure 7D and 7E). Therefore, the results *in vivo* were consistent with our results *in vitro*, which further confirmed that NSUN2 played an important role in enhancing the tumorigenicity of breast cancer cells.

## DISCUSSION

In this study, we showed that the elevated expression of NSUN2 is correlated with the poor prognosis in breast cancer patients. In recent years, the public availability of cancer gene expression data, such as those included in Oncomine and TCGA databases, has provided a unique opportunity to explore initial hypotheses and concepts that can subsequently be validated by additional experiments [22]. Analyzing the data in Oncomine database, we found that NSUN2 expression is upregulated in invasive ductal breast carcinomas, in comparison with the normal tissues, which agrees with the results of previous studies [14, 23]. However, the previous studies, performed using a small number of samples, demonstrated only that NSUN2 is highly expressed in breast cancer cells, and found that the increase in NSUN2 mRNA levels correlates with the copy number gain of NSUN2, while our results showed that NSUN2 is upregulated in breast cancer cells at both mRNA and protein levels, in comparison with normal breast epithelial cells. Differential expression of NSUN2 was found in the matched breast cancer lesions and adjacent non-cancerous tissues, with a significant increase in NSUN2 levels in cancer cells. Furthermore, IHC analysis results indicate that the NSUN2 expression levels are significantly correlated with the clinical stage of the disease. These findings provide strong evidences that the upregulation of NSUN2 plays an important role in progression of breast cancer.

The overexpression of NSUN2 in breast cancer cells has been reported in several studies [14, 23], but the underlying mechanism has remained unidentified. It was shown that tumorigenesis is associated with the progressive accumulation of genetic and epigenetic alterations in genes and proteins that regulate cell proliferation, cell death, and genomic instability [24, 25]. We performed *in silico* analysis using data from UCSC gene browser and TCGA, found a 156 bp long CpG island in NSUN2 promoter region, and showed that this promoter is hypomethylated in breast cancer tissues. Frequent hypomethylation of the NSUN2 promoter region in breast cancer tissues, even in the low-grade tumors, is comparable to the hypomethylation frequencies of known oncogenes in breast and other types of tumors [26]. A systematic analysis of NSUN2 promoter methylation levels in human breast cancer cell lines showed that these levels are lower than that in the normal breast epithelial cells. Additionally, NSUN2 expression was shown to be induced by the treatment with demethylation agent 5′-AZA in the cells with NSUN2 hypermethylation. DNA methylation is a primary epigenetic gene silencing mechanism, which has been widely associated with all stages of cancer development, and specific methylation events can be used as diagnostic and prognostic biomarkers [27, 28]. Notwithstanding, fewer studies have addressed the role of abnormal demethylation in cancer, although hypomethylation of the genome has been increasingly recognized as a cancer-linked trait, including breast cancer as well [29, 30]. To the best of our knowledge, this study is the first to show that NSUN2 gene expression is regulated through the promoter hypomethylation in breast cancer cells, and that NSUN2 overexpression is partly due to DNA demethylation.

The overexpression of NSUN2 was shown to significantly increase cell proliferation, migration, and invasion of breast cancer cells. Conversely, NSUN2 knockdown markedly reduced the proliferation, migration, and invasion of cancer cells *in vitro*, and tumorigenicity *in vivo*. Therefore, *in vivo* results were consistent with our results obtained *in vitro*, further confirming that NSUN2 plays an important role in the tumorigenicity of breast cancer cells. Our findings unveiled the oncogenic role of NSUN2 in the development and progression of breast cancer, and suggest that NSUN2 might be a novel prognostic marker and therapeutic target.

ER-positive breast cancers account for 60-70% of the total number of breast cancer cases, but the remaining cancer cases are ER-negative, they respond poorly to traditional therapies [31], and are more aggressive. PR is present in both normal and malignant cells in breast, and its synthesis depends both on estrogen and ER. Recently, the absence of PR was shown to be an independent predictor of poor response to antiestrogen therapy, and it is associated with higher recurrence rates and shorter survival time [32]. Here, we found that NSUN2 expression is negatively correlated with ER and PR expression levels. These results demonstrate that NSUN2 overexpression in breast cancers tissues is related to ER-negative and PR-negative breast cancer subtypes, but, more importantly, NSUN2 may be a novel target for the development of effective treatment of these aggressive breast cancer types. The treatment of ER-negative or PR-negative breast cancers and, in particular, triple-negative breast cancers is currently very difficult, and therefore, the identification of the suitable cancer biomarkers represents the first step towards the effective individualization of the targeted therapy for these cancers with poor prognosis. Ki-67 is one of the most widely used IHC proliferation markers, which was confirmed as an independent predictive and prognostic factor in the early breast cancers [33]. Ki-67 expression was shown to correlate with NSUN2 expression (*P*<0.001), which agrees with previous studies showing that Ki-67 index correlates with the tumor grade and clinical behavior of cancer [34, 35].

In summary, we showed that NSUN2 is a strong and clinically meaningful biomarker in breast cancer, whose expression correlates with the cancer clinical stage, ER and PR status, and Ki67 expression. Our findings additionally indicate that NSUN2 is involved in breast cancer progression and that it may be used as a potential therapeutic target in breast cancer.

## MATERIALS AND METHODS

### Cell lines

Human epithelial breast cell line, MCF-10A, and breast cancer cell lines, including MDA-MB-231, MDA-MB-435, MDA-MB-453, MCF-7, SK-BR-3, T47D, and BT-549, were obtained from the Cell Bank of the Chinese Academy of Sciences (Beijing, China) between June and November 2015. All cells lines were authenticated using short tandem repeat (STR) method, performed by this cell bank. MCF-10A cells were maintained in Dulbecco's Modified Eagle Medium (DMEM, Thermo Fisher Scientific, Carlsbad, CA), containing 0.5 μg/mL of hydrocortisone, 10 μg/mL insulin, 20 ng/mL human epidermal growth factor (EGF), and 5% (vol/vol) heat inactivated horse serum. Breast cancer cells were maintained in DMEM (ThermoFisher Scientific) supplemented with 10% fetal bovine serum (FBS; ThermoFisher Scientific). All experiments using cells were performed within six months after obtaining these cell lines.

### Patient information and tissue specimens

A total of 191 paraffin-embedded tissues of breast cancer were used in this study, obtained from the patients that were histopathologically and clinically diagnosed at the Peking Union Medical College Hospital (PUMCH), Beijing, China during 2013 and 2014. Tissues were obtained from patients who were subsequently diagnosed with breast cancer, and 10 freshly prepared human breast cancer tissues were frozen and stored in liquid nitrogen until further use. Clinical and clinicopathological cancer classification and staging were performed according to the American Joint Committee on Cancer (AJCC) criteria. Histological tumor grade was determined according to the Elston-Ellis modification of the Scarff-Bloom-Richardson (SBR) system. Clinical information related to the samples is summarized in Table 1. Informed consents were obtained from all patients included in this study, and the study and all procedures used were approved by the Institutional Research Ethics Committee of PUMCH.

### Western blot

Protein lysates were obtained from cells in the sampling buffer [62.5 mmol/L Tris-HCl (pH 6.8), 10% glycerol, 2% SDS], and separated using 10% Tris-glycine gels and SDS-Tris-glycine running buffer, and the separated proteins were blotted onto the nitrocellulose membranes. Membranes were incubated at 4°C overnight with primary polyclonal anti-NSUN2 (Abcam, Cambridge, MA, USA) and polyclonal anti-GAPDH (Abcam, Cambridge, MA, USA) antibodies, which was followed by washing with Tris buffered saline Tween-20 (0.25%, TBS-T), and the incubation with the secondary antibody (goat radish peroxidase-conjugated anti-rabbit IgG; Santa Cruz Biotech, CA, USA) for 1 h at room temperature. Signal detection was performed using ECL Prime system (Pasadena, CA, USA) and GE Imaging System (Pasadena, CA, USA ) according to the manufacturer's instructions.

### Lentiviral construction and stable expression of ectopic genes

NSUN2 expression construct was generated by the subcloning of PCR-amplified full-length human NSUN2 cDNA into the pHBLVCMVIE-IRES-Puro vector (HANBIO Biotechnology, Shanghai, China). To silence endogenous NSUN2, two shRNAs were cloned into the pHBLV-U6-Zsgreen-Puro vector (HANBIO Biotechnology, Shanghai, China), To generate pSuper-retro-NSUN2-RNAi(s), transfection of siRNAs or plasmids was performed using Lipofectamine 2000 reagent (ThermoFisher Scientific, Carlsbad, CA) according to the manufacturer's instructions. Stable cell lines with the overexpressed or silenced NSUN2 were generated using lentiviruses and HEK293T cells, as previously described [36], and selected with 0.5 μg/mL of puromycin for 10 days.

### CCK-8 assay

Cells were seeded in 96-well plates at 2×10^3^/well and cultured for 24, 48, and 72 h. 10 μL CCK-8 (Dojindo, Japan) was added to the cells, and their viability was measured at 490 nm, using an ELISA plate reader (BioTek, Winooski, VT), according to the manufacturer's instructions.

### Colony formation assays

Cells were plated in six-well plates (1×10^3^/well) and cultured for 14 days. The colonies were stained with Giemsa for 30 min, then fixed with methyl alcohol for 30 min. Cells were washed by phosphate buffered saline (PBS), photographed and counted by using a light microscope (Nikon, Tokyo, Japan) to determine colony formation rates.

### BrdU-DNA synthesis assay

DNA synthesis was determined using BrdU assay, which can indicate the cell proliferation rate. Cells were seeded in 96-well plates at 2×10^3^/well, and the colorimetric BrdU Cell Proliferation ELISA Kit (Abcam, Cambridge, MA, USA) was used. BrdU was added to the culture medium for 12 h before the end of the incubation period. Afterward, the cells were fixed, DNA molecules were denatured, and BrdU content was assessed using a monoclonal anti-BrdU antibody, following the manufacturer's instructions.

### Migration and invasion assays

1×10^4^ MCF-7 cells with stable NSUN2 expressions and the control cells, together with MDA-MB-231 cells with stable shNSUN2 expression, and the control cells, were collected, resuspended in 100 μL of basal medium, and transferred to the transwell chambers (Corning, NY, USA). Complete medium (600 μL) was added to the wells and the chambers inserted into the wells. Plates were incubated at 37°C for 24 h, and the remaining cells were swabbed from the top of transwells, and the chambers were submerged in methyl alcohol for 30 min. Cells in the wells were stained with Giemsa for 30 min, washed by PBS, and counted by using the light microscope to determine cell migration. A similar procedure was performed for the invasion assay, with the addition of Matrigel (BD Biosciences, USA) to the wells of 24-well plates. Matrigel was incubated in pre-chilled basal medium (30 μL) at 37°C for 1 h. The excess medium was discarded, and 100 μL of basal medium were added to the well containing Matrigel and 600 μL to the chamber, which was followed by incubation at 37°C overnight. The rest of procedure was the same as the migration assay.

### Ms-PCR for the methylation level of promoter CpG island

Genomic DNA was isolated from 1×10^6^ breast cancer cells using QIAamp DNA Mini Kit (Qiagen, Hilden, Germany). Afterward, 1g of DNA was used to perform bisulfite modification, using the Epitect Bisulfite Kit (Qiagen, Germany), followed by Ms-PCR. Bisulfite sequencing primers for NSUN2 gene were designed according to the standard methods, described previously [37]. NSUN2 primer sequences for methylated and unmethylated fragments are provided in Table 3.

**Table 3:** Primer sequences used Real-time PCR and Ms-PCR (5′-3′)

	Gene	Forward primer	Reverse primer
**Real-time PCR**	NSUN2	GAACTTGCCTGGCACACAAAT	TGCTAACAGCTTCTTGACGACTA
	GAPDH	GACTCATGACCACAGTCCATGC	AGAGGCAGGGATGATGTTCTG
**Ms-PCR**	Unmethylated NSUN2	TGAGTAATTGGGATTATAGATAGGTG	AAAAAATTCATCTAAAAAAAACAAA
	Methylated NSUN2	CGAGTAATTGGGATTATAGATAGGC	AAAAAATTCGTCTAAAAAAAACGAA

### *In vitro* demethylation of genomic DNA

Cell lines were seeded in six-well plates. Demethylating agent 5-AZA (Sigma-Aldrich, St.Louis, MO), dissolved in DMSO, was added to treat cells at the final concentration of 2 μM, while the equivalent amount of DMSO was used as the control. Cells were harvested after treatment of 72 h, and cell lysates were extracted for Western blot.

### RNA extraction and RT-qPCR

Total RNA was prepared from the frozen tissue samples using RNeasy Mini Kit (Qiagen, Germany), according to the manufacturer's instructions. The isolated RNA (2 μg) was reversely transcribed into cDNA, using SuperScript II Reverse Transcriptase (Invitrogen, NY, USA). Afterward, qPCR was performed to determine NSUN2 mRNA expression level in all primary breast tumors relative to the paired normal breast tissue. Data were normalized to the geometric mean of housekeeping gene GAPDH to control for the variability in the expression levels. NSUN2 primers for qPCR were designed using Primer Express v2.0 software tool. The primers, amplifying the region between 72 to 226 bp of NSUN2 (GenBank: NG_028215.1) are provided in Table 3.

### Immunohistochemistry (IHC) analysis

Immunohistochemical assay was done to check protein expression in 191 human breast cancer tissues. In brief, paraffin-embedded specimens were cut into 4μm sections and baked at 65°C for 30 min. The sections were deparaffinized with xylene and rehydrated. Afterward, they were submerged into EDTA antigenic retrieval buffer and microwaved. The sections were treated with 3% hydrogen peroxide in methanol to quench the endogenous peroxidase activity, which was followed by the incubation with 1% bovine serum albumin (BSA) to block nonspecific binding. Rabbit anti-NSUN2 antibody (1:500; Abcam, Cambridge, MA, USA) was incubated with the sections at 4°C overnight. As the negative controls, this antibody was replaced with normal goat serum or blocked with a recombinant NSUN2 polypeptide, by incubation at 4°C overnight before the IHC staining. After washed, the tissue sections were treated with biotinylated anti-rabbit secondary antibody (Abcam, Cambridge, MA, USA), followed by the additional incubation with streptavidin-horseradish peroxidase complex (Abcam, Cambridge, MA, USA). Tissue sections were immersed in 3-amino-9-ethyl carbazole and counterstained with 10% Mayer's hematoxylin solution, dehydrated, and mounted in Crystal Mount (Electron Microscope Sciences, Hatfield, PA). The degree of immunostaining of formalin-fixed paraffin-embedded sections was reviewed and scored independently by two pathologists, based on the proportion of positively stained tumor cells and the intensity of staining [38, 39]. The proportion of tumor cells was scored as follows: 0 (no positive tumor cells), 1 (<10% positive tumor cells), 2 (10-50% positive tumor cells), and 3 (>50% positive tumor cells). The intensity of staining was graded according to the following criteria: 0 (no staining); 1 (weak staining = light yellow), 2 (moderate staining = yellow brown), and 3 (strong staining = brown). The raw data were then converted to IRS by multiplying the quantity and staining intensity scores. The final scores were: 0, 1, 2, 3, 4, 6, and 9, with 0 to 3 considered weak staining, 4 to 7 moderate, and over 8 strong IRS.

### Xenografted tumor model

Female BALB/c-nude mice (4-5 weeks old, 14 to 16 g) were purchased from the Charles River Laboratories of Beijing, and were housed in barrier facilities with a 12h/12h light/dark cycle. All mouse experiments were performed according to the institutional guidelines and were approved by Institutional Animal Care and Use Committee (IACUC). Mice were randomly divided into two groups (n=5/group). For tumor cell implantation, 1×10^7^ MDA-MB-231-shNSUN2 and the control cells in 100 μL of the solution were injected subcutaneously into the mice. Tumors were examined once every 4 days: length, width, and thickness of the tumors were obtained using calipers and tumor volumes were calculated. At day 16 after the injections, animals were euthanized, and tumors were excised and weighed. Tumor volume was obtained by the ellipsoid volume calculation formula: 0.5× (length×width^2^).

### Statistical analysis

All statistical analyses were carried out using the SPSS 17.0 statistical software. Chi-square test was used to analyze the association between NSUN2 expression and the clinicopathologic characteristics. For the results obtained in the experiments using cell lines or animals, analysis of variance (ANOVA) was used. *P*<0.05 was considered statistically significant in all cases.

## SUPPLEMENTARY MATERIALS FIGURES


