# Supplementary material for: Overexpression of NSUN2 by DNA hypomethylation is associated with metastatic progression in human breast cancer
Source: Oncotarget. 2016 Nov 30;8(13):20751–65. doi: 10.18632/oncotarget.10612 (PMC5400542; doi:10.18632/oncotarget.10612)
Supplement: Supplementary file 1 [file oncotarget-08-20751-s001.pdf]

Overexpression of NSUN2 by DNA hypomethylation is associated with metastatic progression in human breast cancer

SUPPLEMENTARY FIGURES

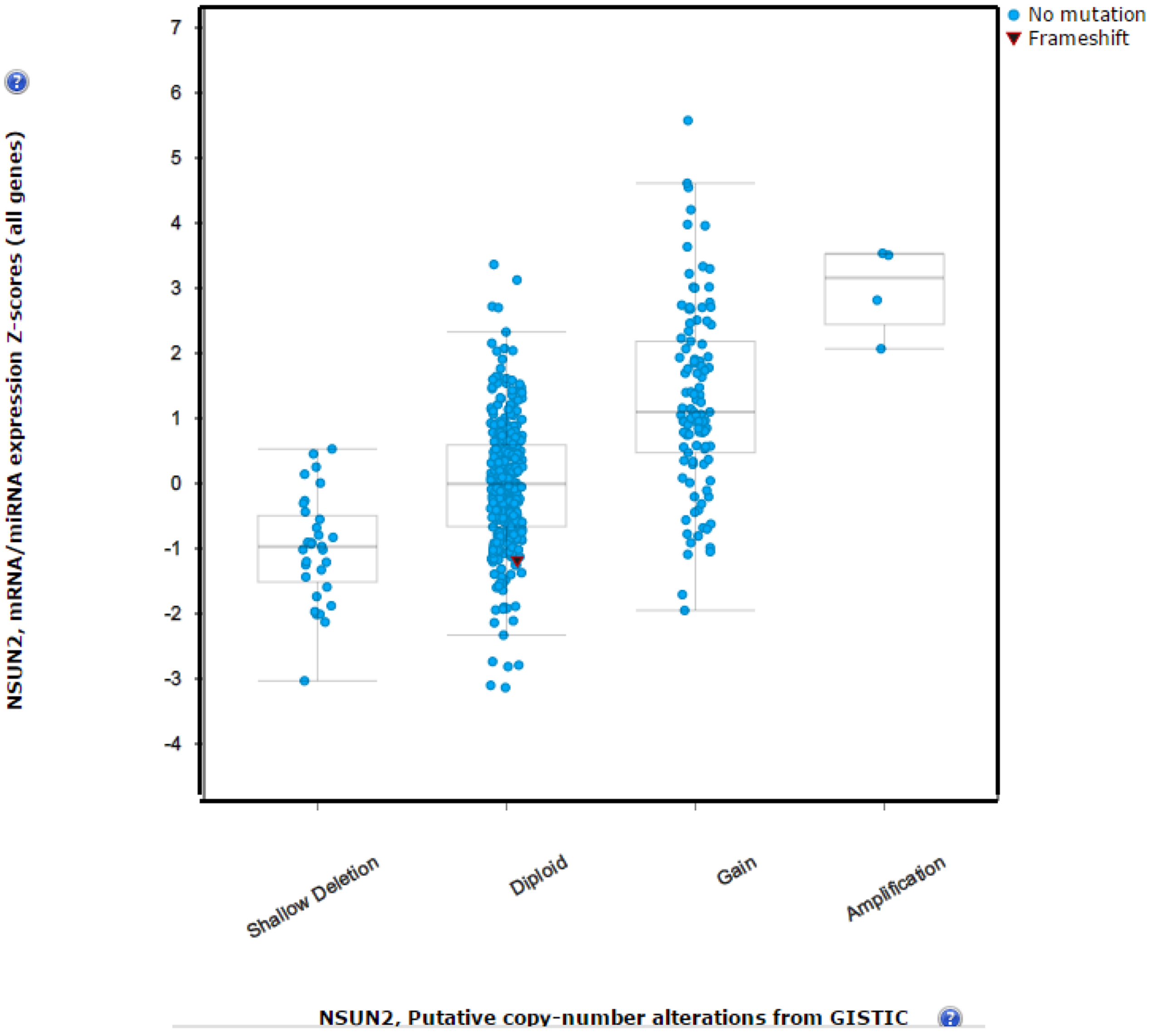

Supplementary Figure 1: NSUN2 mRNA expression is closely related to the changes in NSUN2 copy number.

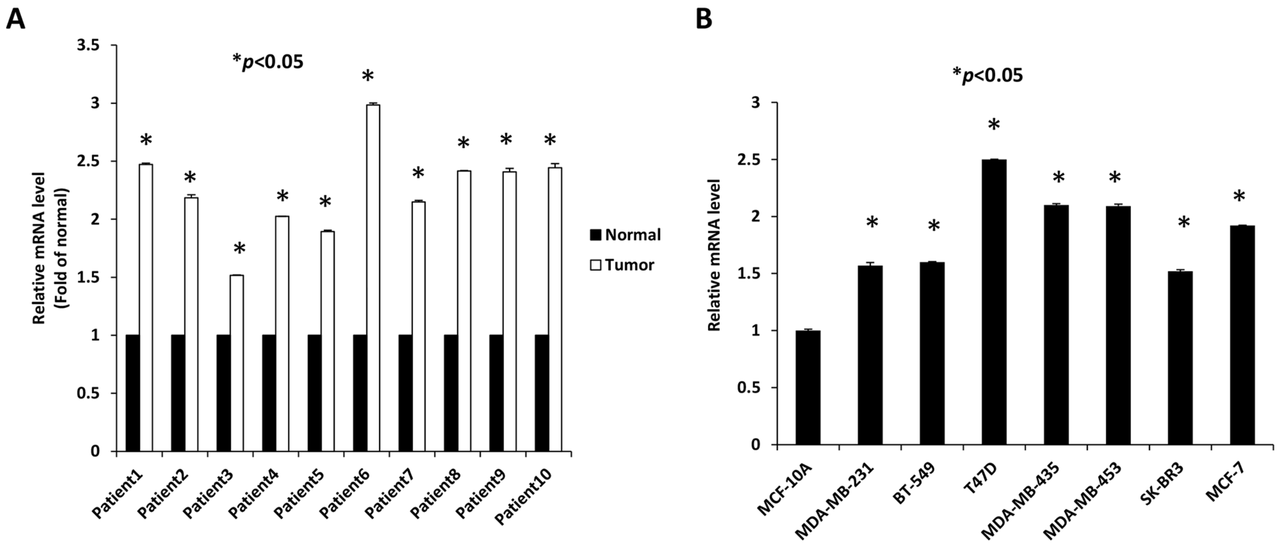

Supplementary Figure 2: NSUN2 mRNA expression in breast cancer tissues and cells. A. NSUN2 mRNA expression in 10 pairs of breast cancer tissues. **B.** NSUN2 mRNA expression in seven breast cancer cells and one normal breast epithelial cell line (MCF-10A). The data from real-time qPCR are represented as mean ± standard deviation (SD) from three independent experiments, *$P<0.05$.

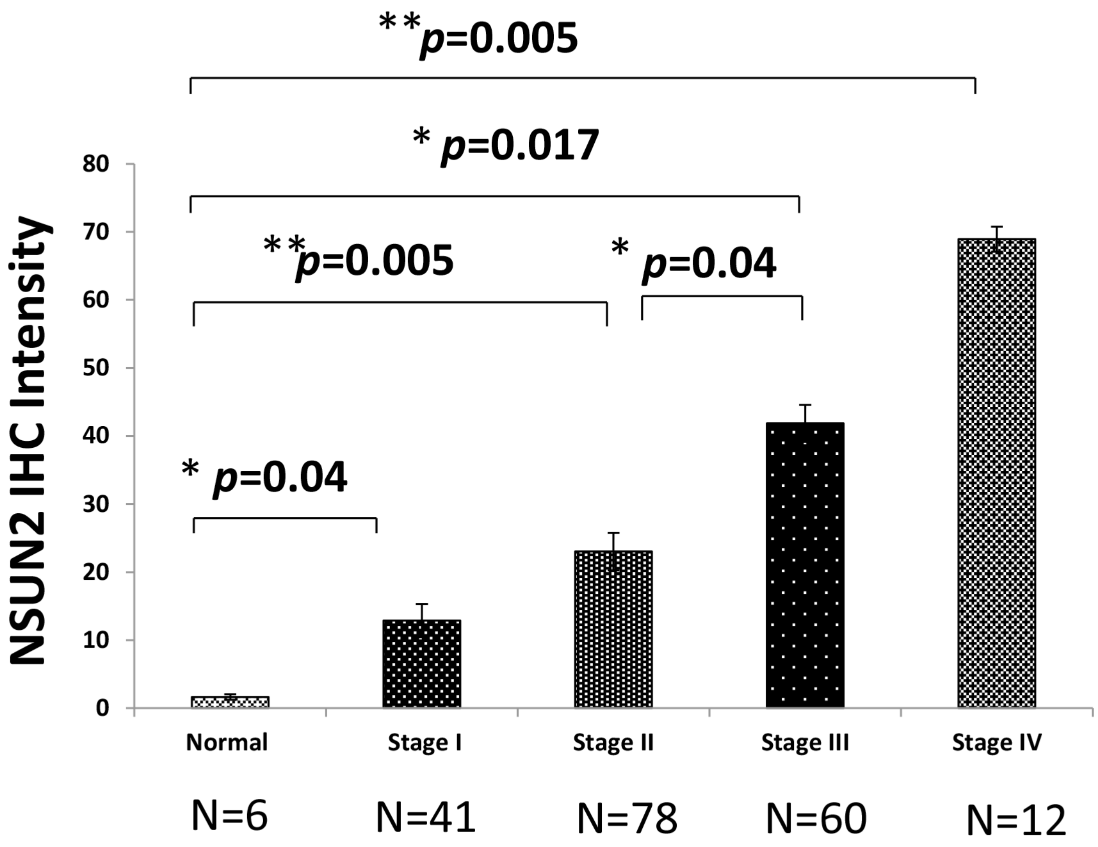

Supplementary Figure 3: NSUN2 protein expression was increased as development of breast cancer. NSUN2 IHC intensity in breast cancer at different stages was shown. In comparison with the normal group, NSUN2 protein expression was shown to be significantly increased in tumor stages I-IV (stage I, P=0.04; stage II, P=0.005; stage III, P=0.017; stage IV, P=0.005). NSUN2 protein expression in stages III and IV was significantly higher than that in stage I (stage III, P=0.04; stage IV, P=0.003). NSUN2 protein expression in stages III and IV was significantly increased, compared with that in stage II (stage III, P=0.003; stage IV, P=0.026). NSUN2 protein expression in tumor stage IV was significantly higher than that in stage III (P=0.0038). Taken together, these results showed the increase in NSUN2 protein expression with the progression of breast cancer. *P<0.05,* *P<0.01.
